# Multifacility outbreak of *Candida auris* during the COVID-19 pandemic—Maricopa County, Arizona, April 2022–December 2022

**DOI:** 10.1017/ash.2023.399

**Published:** 2023-09-29

**Authors:** Siru Prasai, JulieAnna Rivas, Bianca Munoz, Serena Bailey, Karen Zabel, Kaitlyn Chorbi, Rachana Bhattarai, Monica Hernandez-Cubias, Ariella Dale

## Abstract

**Background:** *Candida auris*, an emerging and potentially multidrug-resistant fungus, was first identified in Maricopa County, Arizona, in 2020. On April 21, 2022, an acute-care hospital reported *C. auris* in a bronchoalveolar lavage (BAL) specimen, followed by a second case reported on April 26 identified via retrospective laboratory review and species identification in yeast isolated from a clinical specimen. The Maricopa County Department of Public Health (MCDPH) investigated, and we describe the largest ongoing *C. auris* outbreak containment response in Maricopa County. **Methods:** The MCDPH conducted clinical case and contact investigations in accordance with CDC novel organism containment strategy guidelines. In Maricopa County healthcare facilities (HCFs) with suspected transmission, virtual Infection Control Assessment Responses (ICARs) were administered to identify initial infection prevention and control (IPC) gaps; subsequent regular virtual visits were also provided. HCFs with confirmed transmission completed point-prevalence surveys (PPSs) every 2 weeks until transmission halted as evidenced by 2 sequential negative PPSs. Outreach education to affected HCFs was provided to increase awareness about the public health significance of *C. auris* and the importance of implementation and sustained adherence to standardized IPC protocols. **Results:** In total, 97 HCFs received IPC outreach education, of which 22 HCFs (23%) had suspected transmission and received a virtual ICAR. Contact investigation identified 1,990 contacts, of whom 1,028 (52%) were discharged to the community, 863 (43%) were admitted to other HCFs, and 99 (5%) died. Of the 863 transferred contacts, 10 (1.2%) declined colonization screening, 853 (98.8%) were screened, and 46 (5%) tested positive for *C. auris*. Through sequential PPSs, 101 (5%) of 1,914 screened patients tested positive for *C. auris*. By December 31, 16 clinical and 147 colonized cases were epidemiologically linked to the outbreak. Their median age was 60 years (IQR, 20), and 3 pediatric cases (median age, 17 years) were identified with no pediatric unit admissions. Also, 7colonized cases (5%) developed noninvasive infection and 3 (2%) developed candidemia. **Conclusions:** The MCDPH’s established partnerships with HCFs were key to this ongoing *C. auris* outbreak response spanning 22 facilities over 8 months. Challenges included delays in specimen collection and laboratory processing, operational burden of repeated PPS, and ensuring appropriate precautions for readmitted close contacts at subsequent HCFs. The MCDPH assisted facilities in balancing public health surveillance with facility capacity to execute guidance, including repeated PPS. Consistent adherence to stringent IPC practices, interfacility communication, and proactive *C. auris* education of healthcare workers are paramount to halting transmission.

**Disclosures:** None

